# Anti-nociceptive Activity of Ethnomedicinally Important Analgesic Plant *Isodon rugosus* Wall. ex Benth: Mechanistic Study and Identifications of Bioactive Compounds

**DOI:** 10.3389/fphar.2016.00200

**Published:** 2016-07-06

**Authors:** Anwar Zeb, Sajjad Ahmad, Farhat Ullah, Muhammad Ayaz, Abdul Sadiq

**Affiliations:** Department of Pharmacy, University of MalakandChakdara, Pakistan

**Keywords:** *Isodon rugosus*, ethnomedicine, analgesia, nalaxone, opioidergic receptors, bioactive compounds

## Abstract

*Isodon rugosus* Wall. ex Benth. is extensively used as traditional medicine for the management of various types of pain including tooth ache, gastric pain, abdominal pain, ear ache, and generalized body pain. The current study is designed to scientifically verify the purported uses of *I. rugosus* as analgesic agent and to figure out its possible mechanism of action. Bioactive compounds responsible for analgesic activity were identified using GC and GC-MS analysis. Analgesic potentials were evaluated using acetic acid induced writhing, hot plate test, and formalin induced paw licking test. In acetic acid induced writhing chloroform fraction (Ir.Chf) exhibited 53% analgesia while formalin test displayed 61% inhibition at phase-I and 45% at phase-II respectively at a dose of 100 mg/kg. Similarly, in hot plate test Ir.Chf displayed average reaction time of 7 min at 15, 30, 45, and 60 min intervals. The possible mechanism of action was found to be the central pathway via opioidergic receptors as the mice showed morphine like analgesic activity at pre-administration of naloxone (opioid antagonist) in hot plate and formalin tests. In GC-MS analysis, 83 compounds were identified among which eight compounds including benzyl alcohol, sebacic acid, myristic acid, phytol, sugiol, Tocopherol, α-Amyrin, and stigmasterol were sorted out as previously reported analgesic compounds. Current study revealed that analgesic potential of *I. rugosus* can attributed to the presence of analgesic compounds. It may also be concluded that opioids receptors are involved in the analgesic mechanism of *I. rugosus* due to effective antagonism of nalaxone.

## Introduction

The medicinal use of plants is an ancient tradition, far older than the current sciences of medicine in developing countries. Several herbal remedies are now being intensively used in therapy for different diseased conditions (Ullah F. et al., 2015; Ullah I. et al., 2015; Ayaz et al., 2016). The use of medicinal plants as analgesic drugs in folk medicine is a practice common in developing countries, although in most cases the active principles of the plants are unknown. According to the World Health Organization over 75% of the world's population still depend on plant-derived medicines, usually obtained from traditional healers, for its basic health-care needs (World Health Organization, 1978). Worldwide about 85% of primary health care medications depend on natural sources (Abbasi et al., 2010). Till the nineteenth century, man has discovered the great contribution of herbs in the management of almost every pharmacological disorder (Plummer et al., 2001). According to an estimation, up to 70,000 plant species are used ethno-medicinally worldwide (Haq et al., 2012). According to a survey, medicinal plants cover 34% of the total plant species found in Pakistan (Sher H. et al., 2011; Khan et al., 2016). In Pakistan at least 6000 flowering plants have been reported currently, among which 400–600 are of great medicinal importance (Khan and Khan, 2007). Numerous natural and synthetic compounds have been verified to possess various pharmacological potentials (Ayaz et al., 2015a,b; Kamal et al., 2015a; Sadiq et al., 2015; Ahmad et al., 2016a; Khan et al., 2016). As far as the drugs in the market are concerned, nonsteroidal anti-inflammatory drugs (NSAIDs) are among the most widely used medications due to their efficacy for a wide range of pain, fever and inflammatory conditions as well as cardiovascular protection. However, the long-term administration of NSAID may induce gastro-intestinal ulcers, bleeding, and renal disorders due to their nonselective inhibition of both constitutive (COX-1) and inducible (COX-2) isoforms of the cyclooxygenases enzymes (Halter et al., 2001). It is believed that current pain killer drugs, such as opiates and non-steroidal anti-inflammatory drugs (NSAIDs) are not useful in all cases, because of their various serious side effects and low potency. In this context, a research for other effective alternatives agents is essential and beneficial to reduce the side effects and to treat the concerned diseased condition in a rationalized manner. In the past, search for latest pharmacologically active compounds obtained from natural plants has led to the finding of a number of clinically valuable medicines, such as morphine and aspirin (Calixto et al., 2000; Gilani, 2005). Traditionally in most of part of the world different plant species have been used extensively in the form of paste, powder and decoction for the killing the threshold of pain (Singh et al., 2011). Pain is a condition which is related with every diseased condition but the proper management to reduce or kill the pain in specific health related issues without altering or worsening the side wise diseases.

*Isodon rugosus* Wall. ex Benth. belongs to the family Labiateae. The bark of *I. rugosus* is used ethnomedicinally to treat dysentery and to relieve generalized body pain (Shuaib and Khan, 2015). Locally the dried leaves of this plant is put in mouth as remedy for toothache (Akhtar et al., 2013). The extract of fresh leaves of *I. rugosus* is applied over effected skin for immediate effect and for earache 1−2 drops are used (Sabeen and Ahmad, 2009). It has also been reported to be used ethnomedicinally for gastric and abdominal pain (Ahmad et al., 2014).

Moreover, *I. rugosus* has been used traditionally in the treatment of various infection, blood pressure, pyrexia, rheumatism, microbial infection, and toothache (Khan and Khatoon, 2007; Adnan et al., 2012; Shuaib et al., 2014). This specie has also been verified scientifically to possess pharmacological activities such as hyporglycaemic, anti-diarrheal, and bronchodilator (Sher Z. et al., 2011; Ajmal et al., 2012; Janbaz et al., 2014). Previously, we have reported the preliminary phytochemical analysis and toxicological profile of this plant. Moreover, we have also evaluated the crude extract of this plant for acetyl and butyryl inhibitory and antioxidant potentials which give a preliminary idea about the use of this plant in neurological disorders (Zeb et al., 2014a,b).

Based on the ethnomedicinal uses of *I. rugosus*, the current investigation was undertaken to ascertain the analgesic effects through *in vivo* evaluation of Ir.Chf and to identify its main chemical constituents along with identification of bioactive compounds and validation of its purported medicinal use. The current study is also an attempt to figure out the mechanism of analgesic effect due to suppression of central and peripheral pathways or both.

## Materials and methods

### Plant collection and extraction

The fresh plant was collected from lower Dir (KPK), Pakistan in the month of July and was identified by Dr. Ali Hazrat, plant taxonomist at department of botany, Shaheed Benazir Bhuto University Dir (KPK), Pakistan. The plant sample was kept at the herbarium of the same university with voucher number (1016AZ). Fresh aerial parts of the plant having weight (15 kg) rinsed with uncontaminated water to remove any dust particles and kept in shade for 22 days for drying purpose. The dried plant parts were sliced into small pieces and grinded into coarse powder with the help of a grinder. The powdered material (7 kg) was macerated in 25 liters of 80% methanol for 20 days and extraction with methanol was repeated three times. After soaking, it was filtered using muslin cloth and then through Whattman filter paper. The filtrate obtained was evaporated using rotary evaporator under reduced pressure at 40⋅C (Ahmad et al., 2015; Kamal et al., 2015b; Shah et al., 2015). The filtered solutions were combined and concentrated under reduced pressure using rotary evaporator. A greenish brown semi solid mass of the methanolic extract was obtained having weighing 600 g.

### Fractionation

The crude methanolic extract of *I. rugosus* was transferred into a separating funnel and diluted with 500 ml of distilled water followed by the addition of *n*-hexane (500 ml). The mixture was shaken vigorously and kept for some time to form two layers. The *n*-hexane layer was separated and repeated the same procedure three times by the addition of 500 ml of *n*-hexane each time. All the *n*-hexane fractions were combined and concentrated at reduced pressure using rotary evaporator. The final concentrated weight of *n*-hexane fraction was 27 g. Same procedure was repeated for chloroform, ethyl acetate getting 42, and 94 g respectively and at the end the aqueous fraction was procured weighing 135 g (Ayaz et al., 2014a,b).

### Gas chromatography (GC) analysis

Chloroform fraction was analyzed by means of an Agilent USB-393752 gas chromatograph (Agilent Technologies, Palo Alto, CA, USA) with HHP-5MS 5% phenylmethylsiloxane capillary column (30 m × 0.25 mm × 0.25 μm film thickness; Restek, Bellefonte, PA) equipped with an FID detector. The temperature of oven was preserved at 70⋅C for 1 min at initially, and then gradually increased to 180⋅C at the speed of 6⋅C/min for 5 min and finally increased to 280⋅C at the speed of 5⋅C/min for 20 min. The temperatures of the Injector and detector were set at 220⋅C and 290⋅C, correspondingly. Helium was utilized as carrier gas at a flow rate of 1 ml/min.

### Gas chromatography-mass spectrometry (GC/MS) analysis

GC/MS analysis of the chloroform fraction was processed by means of an Agilent USB-393752 gas chromatograph (Agilent Technologies, Palo Alto, CA, USA) with a HHP-5MS 5% phenylmethylsiloxane capillary column (30 m × 0.25 mm × 0.25 μm film thickness; Restek, Bellefonte, PA) prepared with an Agilent HP-5973 mass selective detector in the electron impact mode (Ionization energy: 70 ev) working under similar experimental environment as illustrated for GC (Ayaz et al., 2015; Ahmad et al., 2016b).

### Identification of components

Compounds present in chloroform fraction of *I. rugosus* were identified based on the comparison of their relative retention indices of each of them with those of authentic compounds in the literature. Additional identification were performed from the spectral data obtained from the Wiley and NIST libraries and further identifications were completed by comparisons of the fragmentation pattern of the mass spectra with the reported literature (Stein et al., 2002; Adams, 2007).

### Experimental animals

The anti-nociceptive evaluation was carried out for which the Swiss albino mice of either sex were obtained from the National Institute of Health, Islamabad, Pakistan. The experimental animals were used with the approval of ethical committee of Department of Pharmacy, University of Malakand, Pakistan according to the animals Bye-Laws 2008 (Scientific procedure Issue-I).

### Acute toxicity test

Albino mice were arranged by dividing into control and test groups each having 5 test models animals. Ir.Chf was administered orally at various doses ranging from 250 to 2000 mg/kg. The solvent used for dose preparation was tween-80. After receiving the doses, the mice were observed for next 72 h for minor allergic symptoms and abnormal behavior (Hosseinzadeh et al., 2000).

### Analgesic activity

#### Acetic acid-induced writhing test

The Acetic acid-induced writhing test was performed for the evaluation of analgesic potential of *I. rugosus*. The Ir.Chf was administered orally at various doses. After 30 min, of interval 0.6% acetic acid (10 ml/kg) was injected intra-peritoneal into the test models (mice). Group I was administered 0.5% tween-80 (3 ml/kg) which served as control, Group II was administered standard drug diclofenac sodium (10 mg/kg) while groups III and IV were administered with Ir.Chf viz., 50 and 100 mg/kg respectively. The number of writhes (contractive movements in the abdomen, twisting of the mice trunk, elongation and extension of body and limbs) were counted from 5 to 30 min after the administration of acetic acid (Franzotti et al., 2000).

#### Formalin test

Swiss albino mice 25–30 g were kept in a temperature controlled environment 23 ± 2⋅C with a 12 h light dark cycle. Food plus water were freely available throughout the experiments. The formalin-induced licking test was performed for the evaluation of analgesic potential of *I. rugosus*. The Ir.Chf was administered orally at various doses. After 30 min, of interval 2.5% formalin (20 μl) (v/v in distilled water) was injected subcutaneously into the plantar surface of the hind paw of the mice. Group I was administered 0.5% tween-80 (3 ml/kg) which served as negative control, Group II was administered standard drug Morphine (5 mg/kg) while groups III and IV were administered with Ir.Chf viz., 50 and 100 mg/kg respectively. The formalin induced licking of the paw was considered as indicative of the nociceptive behavior. The total time spent in the behavioral responses to nociception including licking and biting of injected paw was recorded. The time spent was recorded up to 30 min. The first 5 min was considered as early phase (neurogenic phase) and the second period of 15–30 min as the late phase (inflammatory phase) of the nociceptive response (Sulaiman et al., 2008).

#### Hot plate test

The heated surface of a hot plate (Ugo Basile, model-7280) analgesia meter was maintained at 55 ± 0.2⋅C. The hot plate test was used to measure the response latencies according to the reported procedure (Muhammad et al., 2012). Animals were placed into the glass cylinder on the heated surface, and the time between placement and licking of the hind paws or jumping movements was recorded as response latency which were the parameters evaluated as the thermal reactions. The Ir.Chf (50 and 100 mg/kg, i.p.) and morphine (5 mg/kg, i.p.) were administered 30 min before the beginning of the experiment. Mice were observed before and at 30, 60, and 90 min after Ir.Chf administration. The cut-off time was 20 s.

#### Involvement of opioid receptors

In order to verify the involvement of opioidergic system in the Ir.Chf -induced antinociception, separated groups of mice (*n* = 6) were pre-treated with non-selective opioid receptor antagonist, naloxone (5 mg/kg, S/C), which was injected 15 min before the administration of the Ir.Chf (100 mg/kg; i.p.) and morphine (5 mg/kg), and tested using the hot plate and the formalin test as mentioned above.

## Results

### Gas chromatography-mass spectroscopy (GC-MS)

**Figure 1:**
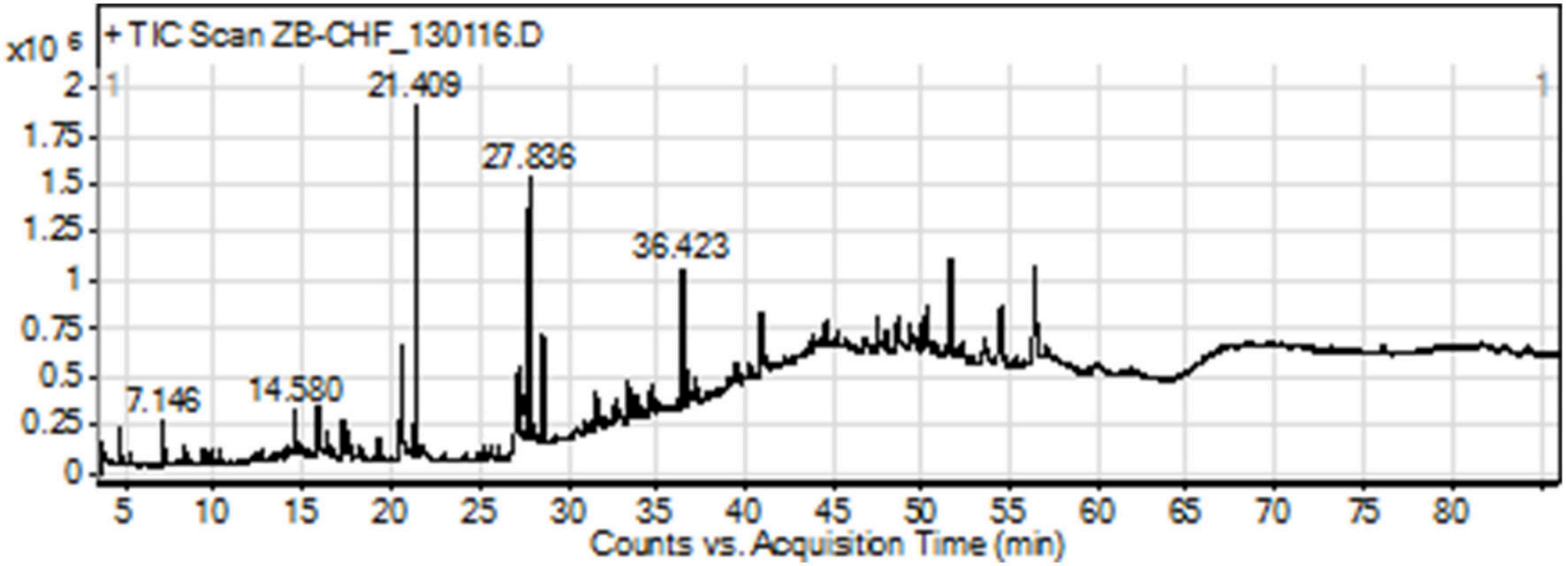
**GC-MS chromatogram of chloroform fraction of *Isodon rugosus***.

The GC-MS analysis of Ir.Chf revealed the identification of 83 compounds. The compounds identified were having the retention time of 3.666–54.438 min. The area-wise highest percentage was shown by ethyl palmitate with retention time 21.411 min, followed by ethyl linoleate and benzenedicarboxylic acid with retention time 27.633 and 36.423 min. The list of all compounds is given in Table 1 and the chromatogram has been shown in Figure 1 in which the peaks are clearly visible. Similarly, the Table 2 shows various parameters of major compounds identified in Ir.Chf.

**Table 1:** **List of compounds identified in the GC-MS analysis of chloroform fraction from *Isodon rugosus***.

**S. No**.	**Compound label**	**RT**	**Name**	**Formula**	**Hits (DB)**
1	(5E)-3,6-Dimethyl-5-octen-2-one	3.666	NF	C10H18O	10
2	2,3-Heptanedione (CAS)	3.865	Acetyl valeryl	C7H12O2	10
3	Methane, sulfonylbis- (CAS)	4.177	Methyl sulfone	C2H6O2S	10
4	Heptenal	4.717	heptenal	C7H12O	10
5	3,5,5-Trimethyl-1-hexene	5.068	NF	C9H18	10
6	2-n-Propylfuran	5.353	2-Propylfuran	C7H10O	10
7	3-(Azidomethyl)cyclohexene	5.582	NF	C7H11N3	7
8	Benzyl alcohol	6.03	Benzyl alcohol	C7H8O	3
9	Butanal, 2-methyl-(CAS) isovaleraldehyde (2-methyl)	6.333	2-Methylbutanal	C5H10O	6
10	N-Butylpropargylamine	7.144	N-Butylpropargylamine	C7H13N	10
11	Octanoic acid, ethyl ester (CAS)	8.406	Ethyl caprylate	C10H20O2	10
12	2-Decenal, (E)- (CAS)	9.41	trans-2-Decenal	C10H18O	10
13	6-Ethyl-5,6-dihydrouracil	9.695	NF	C6H10N2O2	10
14	2,4-Decadienal, (E,E)- (CAS)	10.212	NF	C10H16O	10
15	Pentanal, 3-(hydroxymethyl)-4,4-dimethyl-	10.834	NF	C8H16O2	10
16	Benzaldehyde,3-hydroxy-4-methoxy-(CAS)	11.475	NF	C8H8O3	10
17	9-Oxononanoic acid	12.462	9-Oxononanoic acid	C9H16O3	10
18	Decanoic acid, ethyl ester (CAS)	12.64	Ethyl caprate	C12H24O2	10
19	3-n-Propyl-adamantol-1 $$ 3-Propyl-1-adamantanol #	13.972	NF	C13H22O	10
20	3-Hydroxy-.beta.-damascone	14.079	NF	C13H20O2	1
21	Bicyclo[3.1.1]heptan-3-one, 2-(but-3-enyl)-6,6-dimethyl-	14.15	NF	C13H20O	1
22	1′-Methyl-3′-oxo-3′-(2″,6″,6″-trimethylcyclohex-3″-en-1″-yl)propyl 4-Ethenyl…	14.195	NF	C22H26O3	1
23	2-Cyclohexen-1-one, 4-(3-hydroxy-1-butenyl)-3,5,5-trimethyl-	14.49	NF	C13H20O2	10
24	Decanedioic acid	14.582	Sebacic acid	C10H18O4	10
25	Furan, 2,3-dihydro-2,2-dimethyl-3-(1-methylethenyl)-5-(1-methylethyl)-	14.921	NF	C12H20O	10
26	Pentadecanal-	15.229	Pentadecanal	C15H30O	10
27	4-((1E)-3-Hydroxy-1-propenyl)-2-methoxyphenol	15.866	NF	C10H12O3	10
28	Tetradecanoic acid (CAS)	15.967	Myristic acid	C14H28O2	10
29	Tetradecanoic acid, ethyl ester (CAS) Ethyl-Myristate	16.439	Ethyl myristate	C16H32O2	10
30	3-(2-Methylenecyclopentyl)propyl azide	16.725	NF	C9H15N3	10
31	7,11,15-Trimethyl,3-Methylene-1-Hexadecene	17.317	Neophytadiene	C20H38	10
32	Methyl tridecyl ketone	17.443	2-Pentadecanone	C15H30O	10
33	Hexacosanoic acid, 2,4,6-trimethyl-, 1-methyl ester, (2R,4S,6R)-(-)-	17.848	NF	C30H60O2	10
34	3,7,11,15-Tetramethyl-2-hexadecen-1-ol	18.267	NF	C20H40O	10
35	Decanoic acid, 2,8-dimethyl-, methyl ester (CAS)	18.508	NF	C13H26O2	10
36	1-Dodecanol, 3,7,11-trimethyl-	19.256	Hexa-hydro-farnesol	C15H32O	10
37	Ethyl (2E)-3-(4-hydroxy-3-methoxyphenyl)-2-propenoate	19.447	NF	C12H14O4	7
38	Hexadecanoic acid	20.572	Palmitic acid	C16H32O2	10
39	Ethyl 9-Hexadecenoate	20.697	NF	C18H34O2	10
40	Cyclohexane, 1,2,4,5-tetraethyl-, (1.alpha.,2.alpha.,4.alpha.,5.alpha.)-	21.02	NF	C14H28	4
41	Hexadecanoic acid, ethyl ester (CAS)	21.411	Ethyl palmitate	C18H36O2	10
42	3-[3′,5′-Dimethoxy-4′-hydroxyphenyl]-2-propen-1-ol	21.798	Sinapic Alcohol	C11H14O4	3
43	Gamma.-octadecalactone	24.957	NF	C18H34O2	4
44	Heptadecanoic acid, ethyl ester	25.306	Ethyl heptadecanoate	C19H38O2	10
45	5-[(Trifluoroacetyl)oxy]-decano-lactone	25.65	NF	C12H17F3O4	10
46	2-Hexadecen-1-ol, 3,7,11,15-tetramethyl-, [R-[R^*^,R^*^-(E)]]-	26.067	Phytol	C20H40O	10
47	17-Octadecynoic acid	27.193	NF	C18H32O2	10
48	Linoleic acid ethyl ester	27.633	Mandenol	C20H36O2	10
49	9-Octadecenoic acid, ethyl ester $$ Ethyl (9E)-9-octadecenoate #	27.836	NF	C20H38O2	10
50	ethyl 9-octadecanoate	28.003	NF	C20H38O2	10
51	4-Ethylcyclohexanol,c&t	28.244	4-Ethylcyclohexanol	C8H16O	10
52	Octadecanoic acid, ethyl ester (CAS)	28.625	Ethyl stearate	C20H40O2	10
53	Podocarpa-8,11,13-trien-13-ol, 14-isopropyl-	31.052	Totarol	C20H30O	10
54	13-Octadecenal, (Z)-	32.592	NF	C18H34O	10
55	4,4-Dimethyl-3-(3-methylbut-3-enylidene)-2-methylenebicyclo[4.1.0]heptane	32.829	NF	C15H22	10
56	Eicosanoic acid, ethyl ester	33.489	Ethyl icosanoate	C22H44O2	10
57	3-Hexen-1-ol, 2,5-dimethyl-, acetate, (Z)-	33.798	NF	C10H18O2	10
58	Bicyclo[3.1.1]heptan-3-one, 2,6,6-trimethyl- (CAS)	34.023	NF	C10H16O	10
59	Cyclohexane, 1,1′-(1,4-butanediyl)bis-	34.163	NF	C16H30	10
60	Propenoic acid, 1,7,7-trimethylbicyclo[2.2.1]hept-2-yl ester, exo-	34.757	NF	C13H20O2	1
61	2-Allylcyclododecanone	35.073	2-Allylcyclododecanone	C15H26O	1
62	1-Octadecanol (CAS)	36.16	Sipol S	C18H38O	2
63	1,2-Benzenedicarboxylic acid, bis(2-ethylhexyl) ester (CAS)	36.423	NF	C24H38O4	10
64	12-Hydroxyabieta-8,11,13-trien-7-one	36.606	Sugiol	C20H28O2	2
65	3,7-Dimethyl-4,5,6,9-tetrahydrocoumaran	36.717	NF	C10H16O	10
66	Beta.-Hydroxytotarol	36.943	Totaradiol	C20H30O2	5
67	N-(4-Hydroxy-3-Methoxybenzyl)-8-Methyl-Nonanamide	37.116	NF	C18H29NO3	10
68	3-phenyl-6-trimethylsilyl-pyrazolo[1,5-a]pyridine quinone-2,5	40.139	NF	C16H16N2O2	10
69	Ethyl tetracosanoate	40.443	Ethyl tetracosanoate	C26H52O2	1
70	Chlordehydromethyltestosterone	40.93	NF	C20H27ClO2	4
71	Stigmasteryl tosylate	43.923	Stigmasteryl tosylate	C36H54O3S	8
72	Stigmasta-5,22-dien-3-ol, acetate, (3.beta.)- $$ Stigmasterol acetate	44.451	Stigmasterol acetate	C31H50O2	10
73	N-Pentadecane	44.669	Pentadecane	C15H32	7
74	Cholest-5-ene, 3-bromo-, (3.beta.)-	44.736	NF	C27H45Br	10
75	Vitamin E	45.326	alpha.-Tocopherol	C29H50O2	10
76	stigmasta-5,22-dien-3-ol, (3.beta.,22e)-	47.49	NF	C29H48O	10
77	Hexadecane (CAS)	47.99	Cetane	C16H34	10
78	4,22-Cholestadien-3-one $$ (22Z)-Cholesta-4,22-dien-3-one	50.196	NF	C27H42O	6
79	Olean-12-en-3-ol, (3.beta.)- (CAS)	50.31	.beta.-Amyrin	C30H50O	10
80	4-[(2′-Phenyl-1′,3′-thiazol-4′-yl)methyl]-3-methyl-2,2-bis(trifluoromethyl)-…	50.658	NF	C16H12F6N2O2S	1
81	(cis)-10-Ethyl-3,4-dihydro-5-methoxy-1,3-dimethyl-1H-naphtho[2,3-c]pyran	50.89	NF	C18H22O2	1
82	Stigmast-4-en-3-one	51.625	Sitostenone	C29H48O	10
83	cyclopropa[5,6]stigmast-22-en-3-ol, 3′,6-dihydro-, (3.beta.,5.beta.,6.alpha.	54.438	NF	C30H50O	2

R and R^*^ represent same groups on different positions.

**Table 2:** **Various parameters of the peak list of chloroform sample of *Isodon rugosus***.

**Compounds**	**RT**	**Height**	**Height %**	**Area**	**Area %**	**Area Sum %**	**Base Peak m/z**	**Width**
Heptenal	4.718	184482	10.33	388866	4.61	1.8	83	0.137
N- Butylpropargylamine	7.146	220993	12.37	715669	8.48	3.32	68.1	0.147
Octanoic acid, ethyl ester	8.405	82604	4.62	123205	1.46	0.57	88	0.06
2-Decenal	9.41	87570	4.9	164981	1.95	0.77	70	0.077
2,4-Decadienal	10.213	69191	3.87	217324	2.57	1.01	81	0.111
Decanedioic acid	14.58	161559	9.04	319108	3.78	1.48	55.1	0.067
Hexadecanoic acid, ethyl ester	21.409	2E+06	100	8E+06	100	39.18	88	0.204
Linoleic acid ethyl ester	27.632	1E+06	59.24	4E+06	49.9	19.55	67.1	0.144
9-Octadecenoic acid, ethyl ester	27.836	1E+06	71.75	5E+06	62	24.29	55.1	0.164
1,2-Benzenedicarboxylic acid, bis(2-ethylhexyl) ester	36.423	553876	31	1E+06	16.26	6.37	149	0.087

### Acute toxicity

No mortality and behavioral change was recorded at the specified doses during acute toxicity assay. So, the dose upto 2000 mg/kg was considered as safe for *I. rugosus*.

### Writhing test

The acetic acid induced writhing test for evaluation of analgesic activity demonstrated a dose dependent activity. The mean inhibition of writhes of positive control at the dose of 10 mg/kg was 16.5 ± 0.76 with 73.02% inhibition. The Ir.Chf exhibited mean inhibition of 28.33 ± 0.55 with 53.67% inhibition at the dose of 100 mg/kg while at 10 mg/kg it exhibited mean inhibition of 39.83 ± 0.87 with 34.87% inhibition. The Ir.Chf showed a comparative response (53.67%) with the positive control (73.02%) at the dose of 100 mg/kg as shown in the Table 3.

**Table 3:** **Results of acetic acid induced writhing test**.

**Samples**	**Dose (mg/kg)**	**Mean writhes (SEM)**	**% Analgesic activity**
Negative cont.	—	61.16 ± 0.74	0.00
Ir.Chf	50	39.83 ± 0.87	34.87^***^
Ir.Chf	100	28.33 ± 0.55	53.67^***^
Positive cont.	10	16.5 ± 0.76	73.02^***^

*** *P < 0.001*.

### Formalin test

Intraplantar injection of 2% formalin to the mice model evoked a typical biphasic licking response. The duration of licking for the early phase (0−5 min) was recorded as 56.50 ± 0.76 s and for the late phase (15−30 min) was 78.83 ± 0.70 s in control groups. As shown in Table 4, pre-treatment with different doses (50 and 100 mg/kg i.p.) of Ir.Chf had significant effect and dose-dependent against the duration of licking activity in both phases i.e., doses of 100 mg/kg produced a marked reduction of 61.36 and 45.88% inhibition of paw licking in the early and late phase, respectively. Similarly, morphine (5 mg/kg, i.p.) exhibited marked potential in reduction both the neurogenic pain (early phase, with inhibition of 84.95%) and inflammatory pain (late phase, with inhibition 91.33%) in the formalin test. The Morphine with naloxone exhibited 7.83% activity in the early phase and 14.37% in the late phase. The Ir.Chf with naloxone exhibited 12.99% pain reduction in the early phase and 19.02% pain inhibition in the late phase. In the same way, naloxone reversed significantly the antinociceptive effect of Ir.Chf (100 mg/kg) both in the early and late phase as those of morphine.

**Table 4:** **Effect of chloroform fraction of *Isodon rugosus* on formalin induced pain in mice**.

**Samples**	**Dose (mg/kg)**	**Total time spent in licking**			
		**0–5 min**	**% Inhibition**	**15–30 min**	**% Inhibition**
Negative cont.	—	56.50 ± 0.76	—	78.83 ± 0.70	—
Ir.Chf	50	37.00 ± 0.57	34.51^***^	54.16 ± 0.60	31.29^***^
Ir.Chf	100	21.83 ± 0.60	61.36^***^	42.66 ± 0.84	45.88^***^
Mor	5	08.50 ± 0.76	84.95^***^	06.83 ± 0.60	91.33^***^
Mor + Nal	5 + 1	52.33 ± 0.66	7.38^**^	67.50 ± 0.42	14.37^***^
Ir.Chf + Nal	100 + 1	49.16 ± 0.94	12.99^***^	63.83 ± 0.79	19.02^***^

*Ir.Chf, Chloroform fraction; Mor, Morphine; Nal, Naloxone*.

** P < 0.01;

*** *P < 0.001*.

### Hot plate test

Results of hotplate test are summarized in Table 5. The Ir.Chf was found to exhibit a dose dependent increase in latency time as that of positive control. At initial 15 min, the mean reaction time of two different doses of Ir.Chf i.e., 50 and 100 mg/kg body weight was recorded as 5.28 ± 0.71 and 7.46 ± 0.59 min respectively. At the last interval i.e., 90 min, the mean reaction time of two different doses i.e., 50 and 100 mg/kg body weight were recorded as 4.56 ± 0.49 and 5.63 ± 0.30 min respectively for Ir.Chf. The reaction time at initial 15 min for morphine at a dose of 5 mg/kg was recorded as 11.16 ± 0.94 min and at 90 min it was recorded as 7.36 ± 0.94 min. Similarly, at initial 15 min, the mean reaction time for Ir.Chf plus naloxone (50 + 1 mg/kg body weight) was figured out as 5.51 ± 0.60 min. While at initial 15 min, morphine plus naloxone (5 + 1 mg/kg body weight) the reaction time was recorded as 4.76 ± 0.30 min. There was observed an obvious reduction in reaction time by the administration of naloxone before the Ir.Chf.

**Table 5:** **Results of analgesic activity following hot plate model**.

**Samples**	**Dose (mg/kg)**	**Reaction time on hot plate**				
		**15 min**	**30 min**	**45 min**	**60 min**	**90 min**
Negative cont.	—	03.70 ± 0.42	04.35 ± 0.57	2.93 ± 0.33	2.93 ± 0.50	2.70 ± 0.44
Ir.Chf	50	05.28 ± 0.71	06.67 ± 0.47	6.56 ± 0.60	5.43 ± 0.88	4.56 ± 0.49
Ir.Chf	100	07.46 ± 0.59	07.13 ± 0.70	7.13 ± 0.60	7.20 ± 0.57	5.63 ± 0.30
Mor	5	11.16 ± 0.94	10.46 ± 0.66	9.73 ± 0.88	9.73 ± 0.60	7.36 ± 0.94
Mor + Nal	5 + 1	04.76 ± 0.30	05.96 ± 0.54	3.96 ± 0.40	3.60 ± 0.76	2.90 ± 0.36
Ir.Chf + Nal	100 + 1	05.51 ± 0.60	06.26 ± 0.47	4.60 ± 0.76	3.88 ± 0.31	3.43 ± 0.66

*Ir.Chf, Chloroform fraction; Mor, Morphine; Nal, Naloxone*.

### Involvement of opioid receptors

It is obvious from the results that the Ir.Chf exhibited same activity as that of morphine in both the test models i.e., the hot plate and the formalin. The effect of Ir.Chf was effectively abolished by opioid antagonist (naloxone). In the hot plate test the reaction time was considerably decreased by the administration of naloxone and in the formalin test the paw licking inhibition was effectively reversed by the naloxone, which demonstrated the involvement of opioid receptors in the analgesic pathway of Ir.Chf.

## Discussion

**Figure 2:**
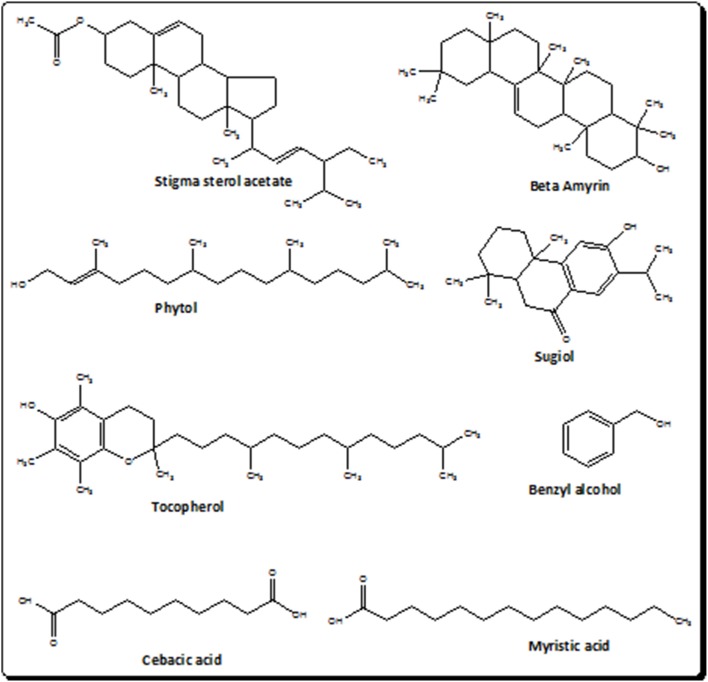
**Structures of bioactive compounds in the chloroform fraction of *Isodon rugosus***.

The current investigational study was carried out to evaluate the antinociceptive effect of *I. rugosus* to verify the ethnomedicinal claims. It was manifested on thermal nociception in hot plate test and chemical nociception in the experimental models of acetic acid-induced writhing and formalin-induced paw licking. The selection of these models was made to investigate the central as well as the peripheral mediated effect of plant sample (Ir.Chf). It has been demonstrated previously that the acetic acid induced writhing involves the peripheral pathway while the hot plate test involves the central pathway in pain mediation. In the same way the formalin test is believed to demonstrate the involvement of both central and peripheral pathways. It has also been previously postulated that acetic acid induce the release of endogenous mediators i.e., prostaglandin E2 and F2α and lipoxygenase products in the intraperitoneal fluids indirectly, which can trigger nociceptive neurons in the proximity. The results of our current investigational study generally suggests the involvement of both central as well as peripheral pathway in the pain suppression as at the dose of 100 mg/kg, Ir.Chf exhibited 53% pain inhibition in acetic acid induced writhing model, 61% activity in formalin induced paw licking model and a wholesome analgesic effect in hot plate model. The result also indicate that the specific pathway involved in the pain suppression is opioidergic pathway, which is depicted from the administration of opioid antagonist (naloxone) to test animals before the administration of Ir.Chf. It was analyzed for molecular characterization which resulted in the identification of 83 different compounds. The compounds identified were analyzed for the presence of bioactive analgesic compounds by literature survey, which revealed the identification of eight compounds viz., benzyl alcohol, sebacic acid, myristic acid, phytol, sugiol, Tocopherol, α-Amyrin, and stigmasterol. The structures of bioactive compounds have been depicted in Figure 2. Briefly, Benzyl alcohol has been verified as an effective local anesthetic. It has also been reported to decreases pain associated with propofol injection (Wilson and Martin, 1999; Minogue and Sun, 2005). Sebacic acid has been reported as an excellent polymer in the case of various drugs formulation that may be analgesics or anesthetics (Shikanov et al., 2007). Similary, the virgin coconut oil has been reported for analgesic activity containing 18.64% of Myristic acid (Intahphuak et al., 2010). In same way, phytol also possess analgesic potential and has been used for antinociception *in vivo* and *in vitro* in various models (Santos et al., 2013). Likewise, the sugiol previously isolated from *Calocedrus formosana* has also been reported for anti-inflammatory activity (Chao et al., 2005). Tocopherol has been reported to be used in neuropathic pain (Kim et al., 2006). α-Amyrin has been verified for antinoception activity in rat models (Pinto et al., 2008). The stigmasterol has also been verified for its analgesic potential (Peres et al., 1998). We can also compare the results of our current investigational study with the previously reported data on *I. rugosus*, in which the tail flick method was followed to figure out the analgesic potential of this plant. In that test the tail deflection time for Ir.Chf and positive control had been recorded as 9.35 ± 0.8 and 9.62 ± 0.2 s respectively, which were almost parallel and comparative (Janbaz et al., 2014). We can observe a similar correlation between the positive control and Ir.Chf in the hot plate test of our current investigational study which is obvious and summarized in Table 4.

## Conclusion

Based on the recorded results and the ethnomedicinal survey of *I. rugosus* Wall. ex Benth. it may be inferred that this plant is a potential source of analgesic compounds, which can produce its analgesic effect due to central analgesic pathway and may be a good candidate in the complementary and alternative medicine. This study provides scientific justification for the ethnomedicinal uses of this plant.

## Author contributions

AZ and SA carried out experimental work, data collection, and literature search under the supervision of AS. FU helped as co-supervision of the research work. MA drafted the manuscript for publication. AS make the final version of publication. All the authors have read and approved the final manuscript for publication.

## Funding

This research received no specific grant from any funding agency in the public, commercial, or not-for-profit sectors.

### Conflict of interest statement

The authors declare that the research was conducted in the absence of any commercial or financial relationships that could be construed as a potential conflict of interest.
